# Mechanical Ventilation-Related High Stretch Mainly Induces Endoplasmic Reticulum Stress and Thus Mediates Inflammation Response in Cultured Human Primary Airway Smooth Muscle Cells

**DOI:** 10.3390/ijms24043811

**Published:** 2023-02-14

**Authors:** Chongxin Yang, Jia Guo, Kai Ni, Kang Wen, Youyuan Qin, Rong Gu, Chunhong Wang, Lei Liu, Yan Pan, Jingjing Li, Mingzhi Luo, Linhong Deng

**Affiliations:** Changzhou Key Laboratory of Respiratory Medical Engineering, Institute of Biomedical Engineering and Health Sciences, School of Medical and Health Engineering, Changzhou University, Changzhou 213164, China

**Keywords:** high stretch, airway smooth muscle cells, mRNA-sequencing, endoplasmic reticulum stress, airway inflammation

## Abstract

Ventilator-induced lung injury (VILI) occurs in mechanically ventilated patients of respiratory disease and is typically characterized by airway inflammation. However, recent studies increasingly indicate that a major cause of VILI may be the excessive mechanical loading such as high stretch (>10% strain) on airway smooth muscle cells (ASMCs) due to mechanical ventilation (MV). Although ASMCs are the primary mechanosensitive cells in airways and contribute to various airway inflammation diseases, it is still unclear how they respond to high stretch and what mediates such a response. Therefore, we used whole genome-wide mRNA-sequencing (mRNA-Seq), bioinformatics, and functional identification to systematically analyze the mRNA expression profiles and signaling pathway enrichment of cultured human ASMCs exposed to high stretch (13% strain), aiming to screen the susceptible signaling pathway through which cells respond to high stretch. The data revealed that in response to high stretch, 111 mRNAs with count ≥100 in ASMCs were significantly differentially expressed (defined as DE-mRNAs). These DE-mRNAs are mainly enriched in endoplasmic reticulum (ER) stress-related signaling pathways. ER stress inhibitor (TUDCA) abolished high-stretch-enhanced mRNA expression of genes associated with ER stress, downstream inflammation signaling, and major inflammatory cytokines. These results demonstrate in a data-driven approach that in ASMCs, high stretch mainly induced ER stress and activated ER stress-related signaling and downstream inflammation response. Therefore, it suggests that ER stress and related signaling pathways in ASMCs may be potential targets for timely diagnosis and intervention of MV-related pulmonary airway diseases such as VILI.

## 1. Introduction

Acute respiratory distress syndrome (ARDS) is a life-threatening acute lung inflammatory process that leads to pulmonary edema, causes severe hypoxemia or critically low arterial oxygen tension, increases lung “stiffness”, and impairs the ability of the lung to eliminate carbon dioxide, necessitating mechanical ventilation (MV) [1]. MV uses a ventilator to provide a positive pressure breath to improve oxygenation of patients [2]. Although MV is an indispensable life-saving support, it can also induce lung injury such as pneumothorax, pneumonia, pulmonary edema, respiratory failure, and even multiorgan failure due to excessive release of inflammatory mediators into the systemic circulation, which is called ventilator-induced lung injury (VILI) [3,4].

Although VILI has been classically attributed to MV-associated high pressure on the respiratory tract, high stretch (excessive mechanical distention) of the airway wall due to MV may also play a crucial role in lung injury since low tidal volume ventilation (reduced airway stretch) has been shown to improve survival of ARDS patients [3,4]. Tidal volume is the amount of air that moves in or out of the lung during each ventilation cycle, which is usually expressed as the ratio of air volume to predicted body weight (mL/kg) [5]. The problem of MV for ARDS patient is that the ventilated lung (referred as “baby lung”) often has decreased volume and compliance due to pulmonary edema and alveolar collapse, resulting in lung inhomogeneity. In this case, higher pressure may be needed for ventilation, but at such high pressure level, a large or even regular tidal volume may cause overdistension of the ventilated lung, inducing volutrauma and barotrauma simultaneously [3,4,6,7]. Therefore, a lowered tidal volume of 4–8 mL/kg is strongly recommended for ARDS patients [8]. However, even though the protective ventilation strategy (limited tidal volume, plateau pressure, and PEEP) has been widely applied in clinical practice, it remains difficult to reduce VILI and ARDS mortality. Therefore, for mechanically ventilated ARDS patients who are perceived to be at the highest risk of death, rescue strategies such as extracorporeal membrane oxygenation, inhaled nitric oxide, prone positioning, and high frequency oscillatory ventilation are often considered, but there is no therapeutic drug to prevent or cure it, suggesting that the underlying mechanism of VILI still needs to be unraveled [6,9,10,11,12,13].

A previous study has shown that high stretch (>10% strain) of cells in the lung during MV induces release of inflammatory mediators from the cells, leading to lung destruction and edema [14]. However, the detail of how high stretch may contribute to the pathological mechanisms of VILI, especially at the level of airway cells, is not well understood. Several pharmacological targets of VILI have also been revealed, which include the blockade of transcription factors and neutralization of inflammatory factors [15]. For example, it has been shown in a C57/BL6 mouse model that DAPK1 mediates apoptosis of alveolar epithelial cells, and inhibition of topoisomerase 1 with topotecan can activate transcriptional response against infection and limit the overexpression of inflammatory cytokines, which all lead to ameliorated VILI [16,17]. In a Sprague–Dawley rat model, it has been shown that inhibition of NF-κB-mediated inflammation by hesperetin, the flavanone subclass of flavonoids, can reduce VILI [18]. However, none of these pharmacological targets seem to be effective in reducing the mortality of VILI in clinical practice [8,19,20].

One area of interest is the role of high stretch in changing cellular behaviors of airway structural cells such as airway smooth muscle cells (ASMCs) and the associated upstream signaling [21,22]. This is because ASMCs are the primary mechanosensitive cells and are known to respond to stretch with changing contractile and secretory phenotypes in airway diseases such as asthma [23,24,25,26]. Therefore, ASMCs may be crucial in mediating airway inflammation in response to high stretch during MV. Conventional hypothesis-driven studies have shown that stretch can modulate the expression of inflammatory factors via certain cellular signaling pathways such as TRPV4 and ERK1/2 [27,28,29]. However, these studies can only identify limited and discrete signaling pathways of interest, which is insufficient to fully understand the stretch-induced responses in a collective and integral perspective, especially the responses associated with the upstream signaling pathways of airway inflammation that are important for timely diagnosis and intervention of VILI.

To overcome this limitation, it may require study of the upstream responding signaling pathways with high throughput screening techniques such as whole genome-wide mRNA sequencing (mRNA-Seq). mRNA-Seq is used to reverse transcribe RNAs of the sample into cDNAs, which are then cloned, amplified and sequenced on the next generation sequencing (NGS) platform based on the technology of simultaneous synthesis and sequencing. This technique uses four kinds of fluorescent-labeled nucleotides to perform parallel sequencing of tens of millions of molecular clusters. When sequencing, fluorescent-labeled reversible termination nucleotides are used, and the sequence and the abundance level of each transcript is determined by reading and analyzing the fluorescence signal [30]. Compared to widely used quantitative (q-PCR) and microarray techniques, mRNA-Seq provides higher coverage and resolution of the transcriptome, which is highly efficient for identifying genes with differential expression under physiological and pathological conditions [31,32].

Other than the conventional hypothesis-driven studies, the whole genome-wide mRNA-Seq studies are data-driven, which are not constrained by established findings and thus may lead to breakthrough insights for cellular response to high stretch. Previously, such an approach has been successfully used to analyze the transcriptomes of biological processes related to stretch-induced lung disease states [33,34]. For example, analysis of mRNA microarray data revealed that L-type voltage-gated calcium channels mediate cyclic stretch (10%, 4 h) and induced inflammatory gene expression in intact bovine trachea smooth muscle [35]. The whole genome-wide RNA-Seq has been used to analyze the expression profiles of circular RNAs (circRNAs) in total lung tissues from a VILI mouse model established using high-tidal volume ventilation, which clarified valuable molecular markers for VILI diagnosis and treatment [36]. However, few studies have used such a method to explore how ASMCs respond to high stretch in terms of mRNA modulation of specific upstream signaling pathways that are involved in VILI.

Therefore, in this study, we systematically evaluated the cellular response of cultured ASMCs to long-term high-stretch treatment (13% strain, 3 days), specifically focusing on the changing profile of mRNA expression in relation to high stretch stimulation via mRNA-Seq, informatics analysis, and functional identification of enriched signaling. Our experimental and analytical results indicate that high stretch mainly induced endoplasmic reticulum (ER) stress and thus activated signaling pathways such as unfolded protein response (UPR) in cultured ASMCs. It is known that ER stress and UPR are involved in many diseases including inflammatory lung disease such as COVID-19, acute lung injury, asthma, chronic obstructive pulmonary disease, and pulmonary fibrosis [37,38,39,40,41], and exposure of cells to mechanical stretch can lead to impaired proteomic homeostasis and thus to accumulation of unfolded or misfolded proteins in ER [42,43]. The unfolded and misfolded proteins in ER will preferentially bind to the heat shock protein family A member 5 (HSPA5), which leads to release of protein kinase RNA-like ER kinase (PERK, also known as EIF2AK3), activating transcription factor-6 (ATF6), and inositol-requiring kinase 1 alpha (IRE1α, also known as ERN1) from the ER membrane. The released EIF2AK3, ATF6, and ERN1 then enhance the expression of HSPA5, X-box binding protein 1 (XBP1), transcription factor 4 (ATF4), and C/EBP homologous protein (CHOP), which all together determine the initiation or mitigation of ER stress, cellular homeostasis, and inflammation response [44,45,46]. Therefore, our data further demonstrate that the high stretch could significantly upregulate the mRNA expression of genes associated with ER stress and inflammation response in cultured ASMCs, and the effects of high stretch on these genes could be completely abolished once ER stress was inhibited. Taken together, these findings provide a novel mechanistic link between mechanical stretch and the functional responses via ER stress in ASMCs, which may serve as a potential therapeutic target for treating high-stretch-induced lung injury such as VILI.

## 2. Results

### 2.1. High Stretch Induces Differential Expression of mRNAs in ASMCs

**Figure 1:**
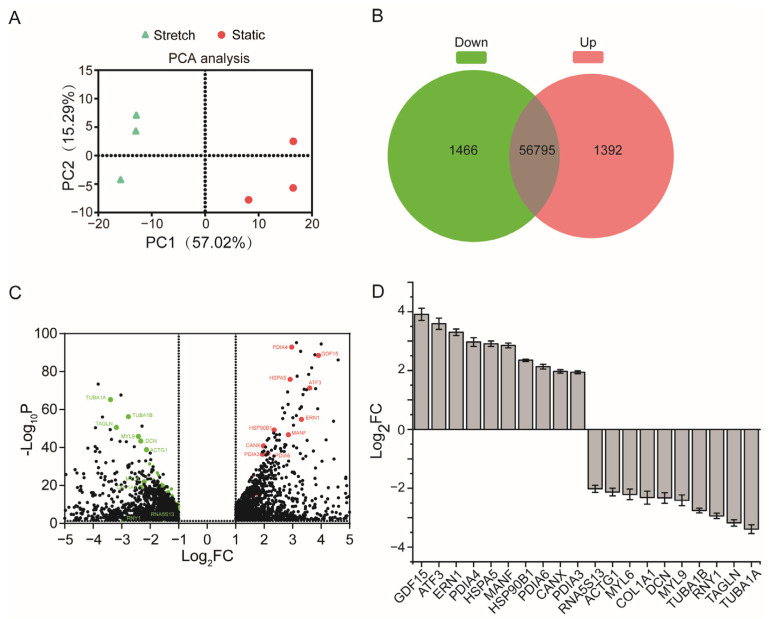
Differentially expressed highly abundant mRNAs in human airway smooth muscle cells (ASMCs) cultured in high stretch (13% strain) compared to the static groups. (**A**) Principal component analysis (PCA) plot of three repeated experiments for ASMCs cultured in static and high-stretch conditions. The first two principal components explained 72.31% of the information (variation) of the dataset (for PC1: 57.02%, for PC2: 15.29%). (**B**) Venn diagram showing overlap of differentially expressed mRNAs (*p* ≤ 0.05 and absolute log_2_ (fold change) >1) in ASMCs between the static and high-stretch groups. (**C**) Volcano plot showing the distribution of differentially expressed mRNAs. Among them, differentially expressed highly abundant mRNAs (DE-mRNAs, i.e., *p* ≤ 0.05, absolute log_2_ (fold change) >1, and count ≥ 100) were shown as red dots (upregulated genes) and green dots (downregulated genes). (**D**) Top 10 DE-mRNAs ranked with fold change in the upregulation and downregulation, respectively. Data are shown as means of three samples. The bars represent the mean ± SEM of mean of 3 independent experiments.

To identify mRNAs in cultured ASMCs that are responsive to mechanical stretch, we performed mRNA profiling using an RNA-Seq and deposited the data to the GEO database (http://www.ncbi.nlm.nih.gov/geo/, GSE206435, accessed on 20 June 2022). PCA analysis as shown in Figure 1A indicates that the expression profiles of mRNAs in ASMCs cultured with or without high stretch were distinctly different, demonstrating a dramatic impact of high stretch on the regulation of mRNAs in the cells. The first two principal components (PC1, PC2) explained 72.31% of the variation of the dataset, or PC1 for 57.02% and PC2 for 15.29%.

The Venn diagram as shown in Figure 1B indicates that in the ASMCs cultured in either high stretch or static conditions, 56,795 genes were overlapped, whereas 2858 genes were differentially expressed, among which 1466 genes were downregulated and 1392 genes were upregulated. The distribution of these differentially expressed mRNAs is shown in Figure 1C as volcano plots, in which their fold change values (log2 scale) were plotted on the *x*-axis against *p* values (negative log10 scale) on the *y*-axis. We then defined the differentially expressed highly abundant mRNAs (DE-mRNAs) in experimental groups as mRNAs with TPM-normalized count ≥ 100 that had an absolute log2 (fold change) expression ≥1 together with a *p* ≤ 0.05 when compared with their counterparts in static cells. It turned out that only a portion of the 2858 differentially expressed mRNAs in the cells could be identified as DE-mRNAs (total 111, 34 upregulated shown as red dots and 77 downregulated shown as green dots in Figure 1C). A complete list of these DE-mRNAs in rank order of fold change was given in Table S1, while the top 10 DE-mRNAs upregulated by high stretch were GDF15, ATF3, ERN1, PDIA4, HSPA5, MANF, HSP90B1, PDIA6, CANX, and PDIA3, and the top 10 DE-mRNAs downregulated by high stretch were TUBA1A, TAGLN, RNY1, TUBA1B, MYL9, DCN, COL1A1, MYL6, ACTG1, and RNA5S13 as shown in Figure 1D.

### 2.2. High-Stretch-Induced DE-mRNAs Are Related to Specific Functional Enrichment

**Figure 2:**
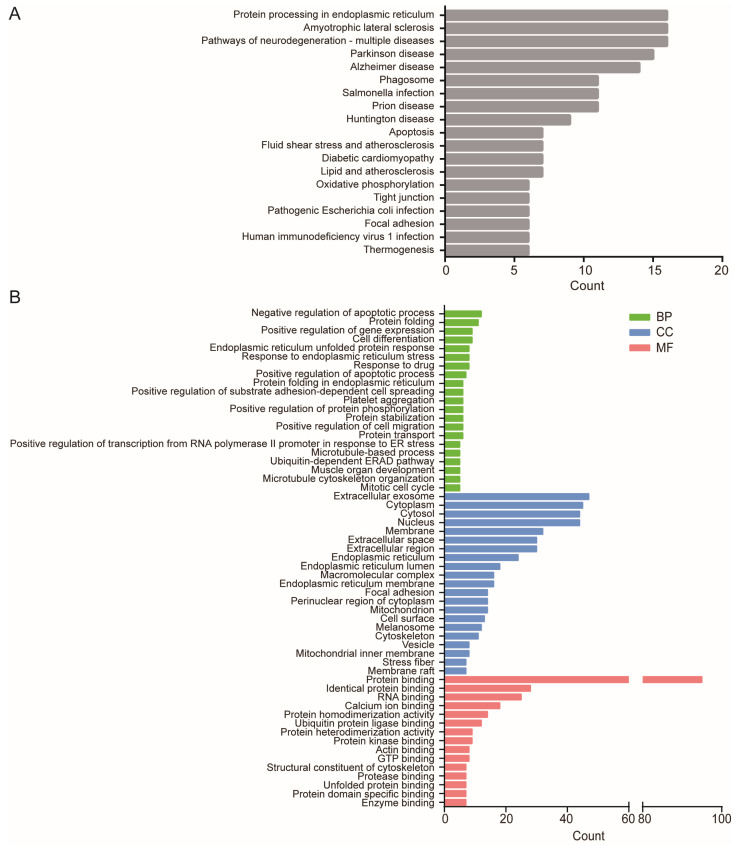
Canonical pathway and gene function enrichment analysis of differentially expressed highly abundant mRNAs with count ≥100 (DE-mRNAs). (**A**) Signaling pathway enrichment by Kyoto Encyclopedia of Genes and Genomes (KEGG) analysis. (**B**) Gene function enrichment by Gene Ontology (GO) analysis in biological process (BP), cellular component (CC) and molecular function (MF). Term is the gene function or signaling pathway description annotation of KEGG or GO, and count is the number of genes enriched in this term.

As displayed in Figure 2A and detailed data in Table S2, KEGG analysis of the 111 DE-mRNAs show that the DE-mRNAs were significantly enriched in protein processing in the endoplasmic reticulum, amyotrophic lateral sclerosis, and pathways of neurodegeneration associated with multiple diseases including Parkinson disease, Alzheimer’s disease, and Huntington disease. Since the chronic neurodegenerative diseases mainly involve misfolded protein processing, these results suggest that ER stress signaling pathways in ASMCs may respond to high stretch by mainly enriching the DE-mRNAs related to misfolded protein processing. Additionally, high stretch appeared to enrich the DE-mRNAs related to cellular mechanics signaling pathways including tight junction and focal adhesion in the cells.

As displayed in Figure 2B and Table S3, GO analysis shows that gene functions of the 111 DE-mRNAs were also significantly enriched in ER stress-related biological processes (BP) including protein folding, endoplasmic reticulum unfolded protein response, protein folding in the endoplasmic reticulum, positive regulation of transcription from RNA polymerase II promoter in response to endoplasmic reticulum stress, and the ubiquitin-dependent ERAD pathway. These DE-mRNAs are also involved in cellular basic behaviors including microtubule cytoskeleton organization, microtubule-based process, positive regulation of substrate adhesion-dependent cell spreading, positive regulation of cell migration, mitotic cell cycle, proliferation, mitosis, and regulation of apoptotic process.

GO analysis also shows that these DE-mRNAs were enriched in cellular components (CC) related to ER stress (incl. endoplasmic reticulum lumen, membrane), cell mechanics (incl. cytoskeleton, stress fiber), and protein transport (incl. extracellular exosome, vesicle, membrane raft), as well as in molecular functions (MF) related to ER stress (incl. protein binding, ubiquitin protein ligase binding, unfolded protein binding), and cell mechanics (incl. actin binding and structural constituent of cytoskeleton).

### 2.3. High-Stretch-Induced DE-mRNAs Forms Distinct Protein–Protein Interaction Network

**Figure 3:**
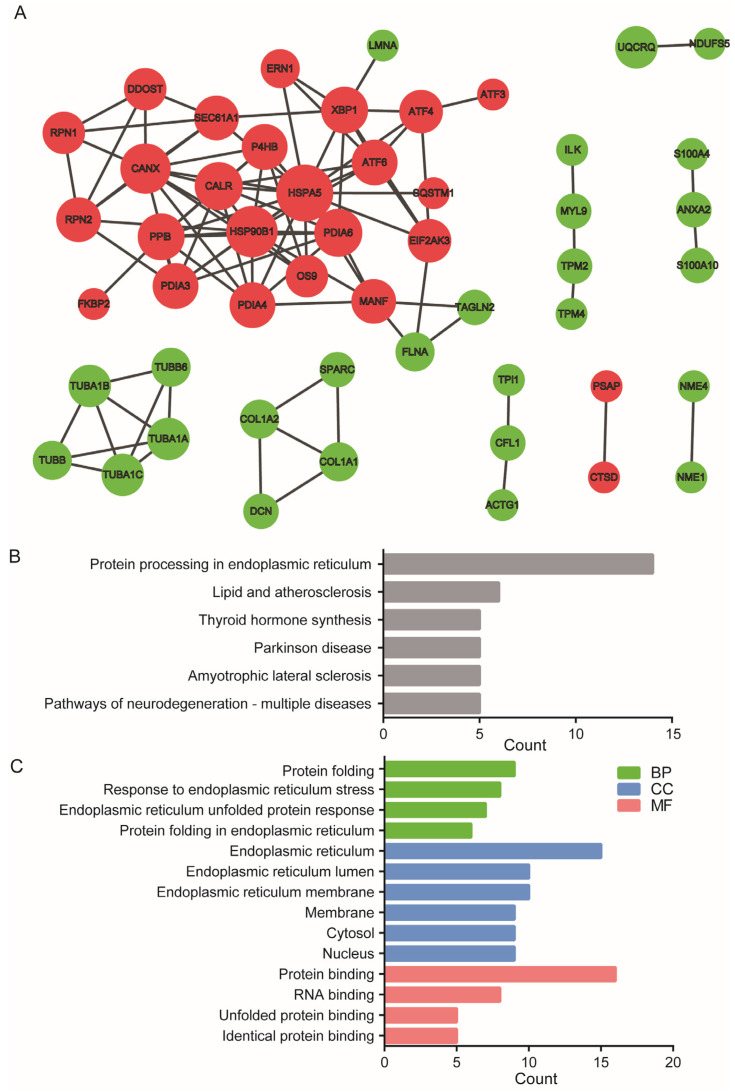
Protein–protein interaction (PPI) networks from differentially expressed highly abundant mRNA with count ≥100 (DE-mRNAs). (**A**) Protein–protein interaction (PPI) networks constructed by using Search Tool for the Retrieval of Interacting Genes/Proteins (STRING) and visualized by Cytoscape. The red nodes stand for upregulated genes, while the green nodes stand for downregulated genes. (**B**) Signaling pathway enrichment by KEGG analysis. (**C**) Gene function enrichment by GO analysis in BP and in CC and MF. Term is the gene function or signaling pathway description annotation of GO, and count is the number of genes enriched in this term.

Based on information from the STRING database, a protein–protein interaction (PPI) network for the targeted proteins of the 111 DE-mRNAs was constructed, which contained 55 nodes and 108 interactions as shown in Figure 3A. Details of the PPI network are described in Table S4. It indicates that the upregulated and downregulated DE-mRNAs formed separate PPI networks, but the network formed with the upregulated DE-mRNAs was the largest, containing 25 nodes.

Subsequently, we defined the DE-mRNAs with interaction degree ≥ 5 in the PPI network as hub genes, which included HSPA5, HSP90B1, CANX, PDIA6, CALR, PPIB, XBP1, P4HB, PDIA3, PDIA4, ATF6, MANF, SEC61A1, RPN2, EIF2AK3, and ATF4 (Table 1). HSPA5, which is a classical marker of ER stress, appeared to be the top hub gene with an interaction degree of 17, while the bottom-ranking hub genes, EIF2AK3 (PERK) and ATF6, are classical ER stress effectors that initiate downstream UPR signaling.

As shown in Figure 3B, KEGG and GO analysis of these 16 hub genes demonstrated that the signaling pathway of the 16 hub genes was mainly enriched in protein processing in the endoplasmic reticulum. Gene function of the 16 hub genes was mainly enriched in BP related to ER stress (including protein folding, response to endoplasmic reticulum stress, endoplasmic reticulum unfolded protein response, protein folding in endoplasmic reticulum in biological process), CC related to ER stress (ER lumen and membrane), and MF related to ER stress (protein binding and unfolded protein binding). These results suggest that ER stress and the related UPR in ASMCs are the most important signaling pathways responsive to high stretch.

### 2.4. Inhibition of ER Stress Prevents High-Stretch-Enhanced Expression of Hub Genes Related to ER Stress and Downstream Inflammation Signaling

**Figure 4:**
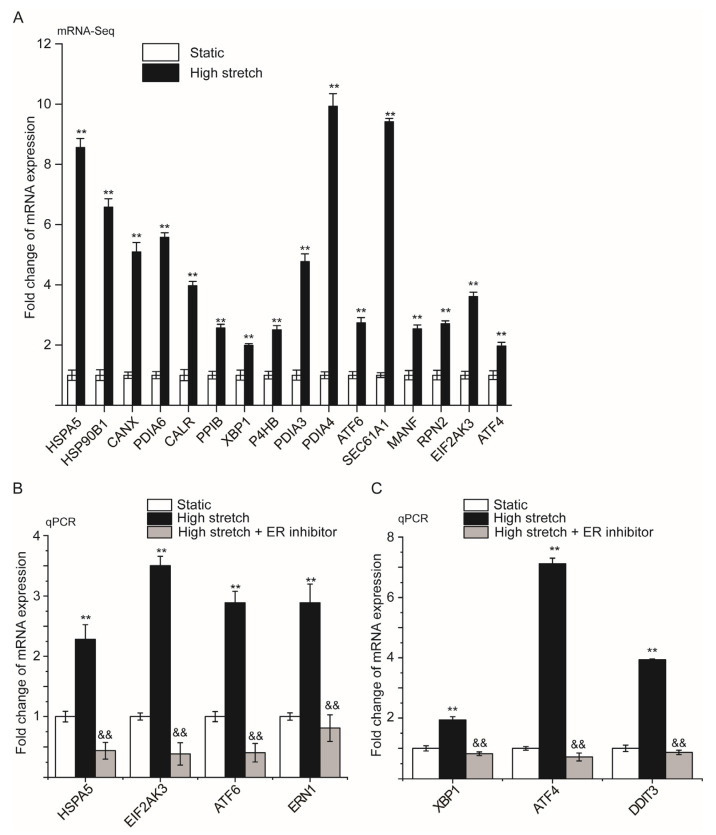
High stretch responses of the hub genes associated with ER stress and the effects of TUDCA on ER stress/inflammation-related signaling in ASMCs. (**A**) Fold changes of mRNA expression of the hub genes associated with ER stress in ASMCs cultured in high stretch (13% strain) compared to the static groups. (**B**,**C**) Fold changes of mRNA expression of genes related to ER stress, and inflammation in ASMCs cultured in high stretch (13% strain) compared to the static groups with or without TUDCA (ER stress inhibitor). n = 3, ** *p* < 0.01, compared to static groups. ^&&^ *p* < 0.01, compared to high stretch groups.

Figure 4A shows the fold change in mRNA expression of the hub genes in ASMCs cultured under high stretch versus static conditions detected by mRNA-Seq. The data show that high stretch enhanced expression levels of all the hub genes. Considering the hub genes mainly involved in ER stress, these results further support that high stretch induced ER stress and related UPR signaling.

On the other hand, we evaluated the effect of TUDCA (a known inhibitor of ER stress) on the primary marker and three main effectors of high-stretch-induced ER stress, namely HSPA5, EIF2AK3, ATF6, and ERN1 in terms of their mRNA expressions in ASMCs cultured under high-stretch condition. The results shown in Figure 4B indicate that in the presence of TUDCA, the high-stretch-enhanced mRNA expression of HSPA5, EIF2AK3, ATF6, and ERN1 were completely prevented, corroborating that the response of ASMCs to high stretch depended on ER stress signaling.

The presence of TUDCA also blocked the high-stretch-enhanced mRNA expression of XBP1, ATF4, and DDIT3 in ASMCs cultured under high-stretch condition as shown in Figure 4C. Since XBP1, ATF4, and DDIT3 are associated with inflammation, these results indicate that the high-stretch-induced responses in ASMCs were also mediated through ER stress downstream inflammation signaling.

### 2.5. Inhibition of ER Stress Prevents High-Stretch-Enhanced mRNA Expression of Airway Inflammatory Factors

**Figure 5:**
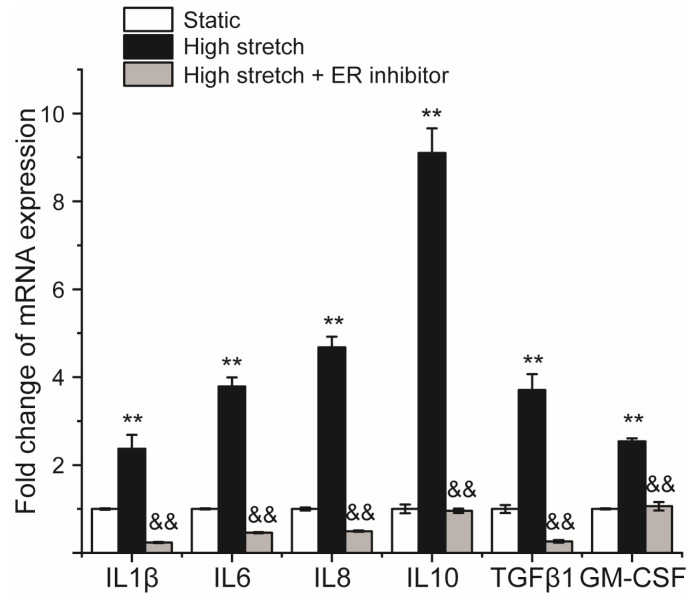
Fold changes of mRNA expression of inflammation factors of ASMCs cultured in high stretch (13% strain) compared to the static groups with or without TUDCA (ER stress inhibitor). n = 3, * *p* ≤ 0.05, ** *p* ≤ 0.01, compared to the static groups. ^&^ *p* ≤ 0.05, ^&&^ *p* ≤ 0.01, compared to the high stretch groups.

Figure 5 shows the fold change in mRNA expression of airway inflammatory factors (IL1β, IL13, IL6, IL8, IL10, TGFβ1, and GM-CSF) in ASMCs cultured under high stretch in the presence or absence of TUDCA. The results indicate that in the absence of TUDCA, high stretch significantly increased the mRNA expression of IL1β, IL13, IL6, IL8, IL10, and GM-CSF in the cultured ASMCs. However, in the presence of TUDCA, the high-stretch-enhanced mRNA expression of these airway inflammatory factors in ASMCs were completely abolished. These data suggest that ER stress response also mediated the high-stretch-enhanced expression of inflammatory factors in ASMCs.

## 3. Discussion

In this study, we aimed to provide insight into high-stretch-induced changes in mRNA expression of ASMCs at a genome-wide scale. The primary finding is that when cultured human ASMCs were exposed to high stretch (13% strain) for a long period (3 days), a panel of 111 mRNAs were differentially expressed with high abundance (count ≥ 100, referred as DE-mRNAs), and 16 of them were hub genes as identified according to their coding proteins’ interaction degree in the PPI network (≥5). More importantly, we found that these hub genes were mainly enriched in signaling related to endoplasmic reticulum (ER) stress, in addition to cellular structure and function. Furthermore, we revealed that active ER stress signaling mediated the high-stretch-enhanced mRNA expression of key factors associated with ER stress and inflammation response signaling in ASMCs. This novel evidence implies that ER stress is probably the most essential response in ASMCs to high stretch through which the secretion phenotype of ASMCs may be regulated, and therefore, it may be a potential alternative theranostic target for timely diagnosis and therapy of high-stretch-related lung disease such as VILI.

To the best of our best knowledge, this is the first attempt to use genome-wide mRNA-Seq and bioinformatics analysis to study the effects of high stretch on ASMCs with a comprehensive perspective of the mRNA expression profile and related signaling pathways in the cells. mRNA-Seq based on next-generation sequencing (NGS) is a high-throughput technology that is widely used to reveal the identity and quantity of RNAs in a given sample. Unlike q-PCR and microarray techniques, mRNA-Seq can be performed without prior knowledge of reference sequences, which enables a wide range of applications including whole genome transcriptome analysis, abundance estimation, and detection of alternative splicing events [47]. This data-driven approach is also not constrained by established models and thus is increasingly adapted in the discovery of new mechanisms of various diseases. All of these advantages can be employed to revolutionize our understanding of the response of ASMCs to high stretch.

Our results show that although ASMCs responded to high stretch by changing cell mechanics-related structure (such as cytoskeleton, tight junction, and cell adhesion) and behaviors (cell division, proliferation, and migration) as known before, the main response of ASMCs to high stretch was ER stress signaling, at least in the case of our in vitro culture. This is somewhat surprising because it has been widely recognized that cell response to mechanical stimulation such as stretch is mainly mediated through conventional signaling pathways such as ion channels, Rho and ERK activation [27,28]. The reason perhaps is that in this study, we applied high stretch to the cultured ASMCs for a longer duration (3 days) than in previous similar studies, which was adopted to mimic the pathological condition of VILI that usually occurs after 3–6 days MV [48]. In this case, the persistent high stretch may continuously stimulate ER stress signaling and eventually lead to dysfunction of proteomic homeostasis controlled by protein production and trafficking that needs a long time to facilitate.

This high stretch can be responded by ASMCs via integrated stress response, such as ER stress triggered by accumulated misfolded proteins [49]. ER stress can be sensed by HSPA5, which in turn activates three main ER stress effectors including EIF2AK3, ERN1 and ATF6 to inhibit general protein expression and thus promote protein misfolding or degrading. In this study using bioinformatics analysis, we confirmed that HSPA5, EIF2AK3, ERN1 and ATF6 were enhanced by high stretch. These results suggest that ER stress-related UPR signaling in ASMCs may be activated by high stretch, which causes disruption of ER function and then accumulation of misfolded and aggregated proteins in the ER lumen, and eventually initiates UPR to promote cellular adaption to changing mechanical conditions.

Although suitable ER stress serves as a feedback mechanism to restore ER homeostasis, prolonged ER stress can also lead to activation of inflammation response. For example, during prolonged ER stress, HSPA5 is triggered to release the effector EIF2AK3. Once the released EIF2AK3 is activated, and subsequently phosphorylates the α-subunit of eukaryotic initiation factor-2a (p-EIF2a), it selectively enhances expression of ATF4. Both ATF4 and cleaved ATF6 then translocate into the nucleus and initiate the transcription of target genes such as CHOP and then initiate an inflammatory response. Furthermore, high stretch can directly induce high production and secretion of inflammatory factors such as IL-6, IL-8 and IL-10, as demonstrated both in vitro and in vivo from cultured airway cells to mechanically ventilated animal models and patients, and these cytokines have been implicated in amplifying lung injury in patients with ARDS under mechanical ventilation [50]. Therefore, pharmacological targeting of this inflammatory cascade at different stages of response by either blockade of transcription factor (NF-kB and AP-1) or neutralization of pro-inflammatory factors (TNF, IL-6, and IL-8) may provide potential therapeutic strategies for treatment of VILI [15]. For example, antibody interventions of IL-8, IL-1, TNF and sFAsL in vitro or in animal models have shown promising therapeutic effects on VILI [51,52,53,54], but these strategies have not demonstrated any beneficial effects on VILI in clinical trials [14,15,55,56].

ER stress has been described in inflammatory airway diseases such as asthma, chronic obstructive pulmonary disease, cystic fibrosis, and VILI [44]. For example, it has been shown that in the stretched alveolar epithelial cells of a VILI rat model, the ER-stress-related EIF2AK3/ATF4 signaling is activated, which can be prevented by an inhibitor of EIF2AK3 (GSK2606414) [57]. In other studies using VILI mouse models, it has been observed that high stretch induces the expression of ER stress markers HSPA5, and inhibitors of ER stress and IRE1α kinase significantly improved VILI [58,59]. In this study, we confirmed that ER stress was enhanced in ASMCs cultured in vitro under high stretch condition, which was largely attenuated or completely abolished by inhibition of ER stress. These data-driven findings provide strong support for the primary role of ER stress in VILI and thus the therapeutic strategy of modulating ER stress response in preventing high-stretch-related lung injury, especially the risk of ARDS in mechanically ventilated patients [60].

In addition to the therapeutic strategy, ER stress biomarkers, especially those that prime the inflammatory response during high stretch, may also be very useful for timely diagnosis of VILI. For example, the ER stress biomarker, HSPA5, is known to bind to immunoglobulin or glucose regulating protein and is the master chaperone protein for UPR in ER stress. When cells are exposed to high stretch, HSPA5 translocates from the ER to the nucleus and the mitochondria, then migrates to the cell surface, and eventually secretes into the circulation via exosomes [61,62]. Considering this translocation and the increased expression of HSPA5 due to high stretch as found in this study, it is quite possible that HSPA5 may provide a potential diagnosis marker of high-stretch-related lung injury such as VILI.

Recent studies on COVID-19 have identified HSPA5 as a potential host–cell receptor for SARS-CoV-2, since lung tissue with high expression of HSPA5 seemed to be highly susceptible to invasion of the virus [63]. Therefore, mechanically ventilated COVID-19 patients may be exposed to greater viral infection due to high-stretch-induced increases in HSPA5 and virus proliferation in the lungs. This probably contributes to the high mortality rate of the critically ill COVID-19 patients who are subjected to chronic mechanical ventilation.

Furthermore, it is known that ER stress is initiated by accumulation of misfolded proteins in ER because, if not immediately disposed, the misfolded proteins in ER often clump together to form aggregates. These aggregates are energetically stable but are non-functional, which places stress on the protein quality control machinery of ER. Normally, the misfolding and aggregation of proteins are detected by stress sensors in ER and properly respond with activation of stress-responsive signaling pathways such as UPR. However, in some cases, the misfolding and aggregation of proteins can overwhelm this system and thus disrupt the cell function [64]. Therefore, it is essential to better understand the underlying mechanisms that influence, control and prevent protein misfolding and aggregation in ER in order to improve and protect ER-stress-related lung disease such as VILI.

Misfolding and aggregation of proteins related to ER stress can be modulated by mechanical factors via different mechanisms [65]. Since ER is an important intracellular Ca^2+^ store, it has been proposed that intracellular Ca^2+^ signaling modulated by mechanical stress initiates ER stress [66]. In advanced heart failure, the cardiac markers of ER stress and cell death can be improved by mechanical unloading with left ventricular assisted devices (LVAD), which is mediated by improved calcium handling [67]. It is also widely believed that shear stress arising from fluid flow can affect protein stability because the shear stress due to a large velocity gradient is theoretically able to induce conformational transition or even unfolding of a protein molecule, which may change the protein’s enzyme activity, molecular aggregation or even its covalent backbone. For example, von Willebrand factor (vWF) is known to unfold under shear force > 30 dyn/cm**^2^**, and the protein extends its polymer chain in orientation to the shear stress direction in favor of self-aggregation [68]. Moreover, it is widely thought that mechanical stress-induced conformational change of buried binding sites in proteins may be a general mechanism for biological mechanosensing [69]. Additionally, it has been proposed that mechanical stress may disturb the cellular quality control system and the molecular chaperones that target misfolded proteins, which may act as a potential driving force for protein aggregation [70]. Therefore, the contribution of mechanical factors to the high-stretch-induced misfolding and aggregation relating to ER stress in ASMCs may be far more complex than the current study demonstrates.

This study is also limited in exploring the mechanism by which high stretch initiates ER stress response. For example, it is known that in the ER membrane, there are various ER stress sensors (such as IRE1a, EIF2AK3, splicing of ATF6), which will be phosphorylated once ER stress occurs and subsequently initiates the following downstream signaling to change the expression of target genes to enhance UPR signaling such as ATF6, XBP1 and CHOP. Therefore, it is important to measure the activation status of these sensors and the protein level of the target genes. However, the exploration of these aspects is beyond the scope of the present study that focused on screening key signals in ASMCs treated with high stretch for 3 days through RNA sequencing and bioinformatics analysis, and these important questions would certainly be addressed in our future studies. Furthermore, our revelation that HSPA5 mRNA expression in cultured ASMCs was enhanced in response to high stretch remains to be determined in circulation and alveolar lavage fluid in high-stretch-related animal models and VILI patients. Such observational studies in animal models and human subjects are critical in further evaluation of the potential of HSPA5 expression as a diagnostic and prognostic biomarker. Besides ASMCs, epithelial cells and macrophages in the airways may also be involved in high-stretch-induced ER stress signaling. Thus, it is important to explore whether the ASMCs-released cytokines would prime the release of cytokines from airway epithelial cells and macrophages by using single-cell sequencing techniques in animal models. In addition, it is also worth pursuing in future studies the therapeutic possibility of mitigating ER stress with chemical chaperones such as 4-phenylbutyrate to reduce lung injury and even multiple organ dysfunction associated with VILI.

## 4. Materials and Methods

### 4.1. Materials

Transferrin (#T8158) and insulin (#91077C) were purchased from Sigma-Aldrich (St. Louis, MO, USA). Collagen type I (#08-115) was purchased from Advanced BioMatrix (#5279, Poway, CA, USA). Dulbecco’s modified Eagle’s medium (#11885092, DMEM), fetal bovine serum (FBS, #16000-044), penicillin–streptomycin (#15140122) and trypsin (#25200056) were purchased from Thermo Fisher Scientific (Waltham, MA, USA). Tauroursodeoxycholic acid (TUDCA, an ER stress inhibitor) was purchased from Solarbio (#IT0790, Beijing, China). Cell culture flasks (#3073) and plates (#CLS3506) were purchased from Corning Incorporated (Corning, NY, USA). All other reagents were purchased from Fisher Scientific unless noted otherwise.

### 4.2. Culture of ASMCs with/out High Stretch

**Figure 6:**
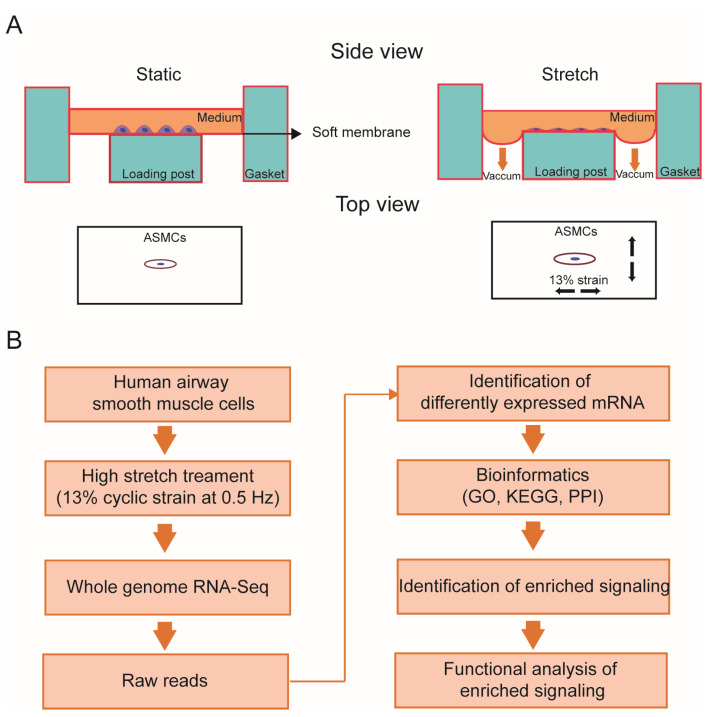
Schematic diagram of the steps to identify high-stretch response signaling. (**A**) Human airway smooth muscle cells (ASMCs) cultured in static (left) and high-stretch (right, 13% cyclic strain, 0.5 Hz) conditions. (**B**) Processes of whole genome-wide RNA-Seq, bioinformatics analysis and functional identification of enriched signaling.

Primary human ASMCs (#BNCC339826) were purchased from BeNa Culture Collection (Beijing, China) and cultured in DMEM supplemented with 10% FBS, 2 mM L-glutamine, 100 units/mL penicillin, and 100 μg/mL streptomycin in an incubator containing 5% CO2 humidified at 37 °C according to the method descripted previously [22,71,72]. ASMCs were identified with an anti-alpha-smooth muscle actin (α-SMA, ASMC marker) antibody (#BM0002, Boster Biological Technology Co., Ltd., Wuhan, China). The cells at a passage of 3–10 were used for experiments since these cells have a typical “hill and valley” appearance under phase contrast microscopy, abundance of α-SMA expression, and physiological contractile functions, all indicating that the cells are a proper phenotype for mechanical experiments. Prior to experiment, exponential proliferating ASMCs (2 × 10^4^ cells/cm^2^) were plated on type I collagen-coated Bioflex 6-well plates and were serum-deprived for 24 h. Since the stretch of airway walls in normal lungs during tidal respiration is estimated to be about 5% and the stretch in ARDS with MV can be twice as large [73,74,75,76], we thus exposed ASMCs grown on Bioflex plates to a 13% cyclic strain at 0.5 Hz for 72 h (a sinusoidal wave, 1 s of deformation alternating with 1 s of relaxation, Flexcell 5000, FlexCell International, Hillsborough, NC, USA) to simulate high stretch on the ASMCs in ARDS during MV [22]. ASMCs grown in Bioflex plates in static condition for 72 h were used as controls. After high stretch treatment, mRNAs were collected and analyzed with whole genome-wide mRNA-Seq or quantitative PCR (qPCR), bioinformatics analysis, and function identification of enriched signaling molecules. A schematic diagram of this study protocol is shown in Figure 6.

### 4.3. Whole Genome-Wide mRNA Sequencing in Cultured ASMCs

mRNA-Seq has emerged as an attractive approach for transcriptome profiling in particular for detecting differentially expressed genes between groups due to its ever increasing high-throughput and decreasing cost for next-generation sequencing [77].

Generally, mRNA-Seq is commonly adopted in four steps. First, sampled RNAs are fragmented and converted into small complementary DNA sequences (cDNA) and then sequenced through a high throughput platform. Second, the small generated sequences are mapped to a transcriptome. Third, the expression level of each gene or isoform is estimated. Fourth, the mapped data are normalized by using statistical and machine learning methods, and the differentially expressed genes are thus identified [78].

To screen signaling pathways in ASMCs responding to high stretch specifically, whole genome wide mRNAs in ASMCs cultured in static and high-stretch conditions were evaluated by using an mRNA-Seq method, which was modified from previous reports [79,80,81]. Briefly, total RNA of ASMCs from either high-stretch or static groups (3 biological replicates each group) was purified using the RNAiso™ Plus Kit (TaKaRa, Japan). After RNA purification and DNase I digestion, ribosomal RNAs were removed from total RNA with the RiboMinusEukaryote Kit (Qiagen, Valencia, CA). The integrity of remaining RNAs was measured by visualization of the 28S and 18S RNA transcripts on a 1.2% agarose, and the quality of RNA was assessed using an Agilent 2100 Bioanalyzer (Agilent Technologies, USA). Then, total RNA was quantified on Nanodrop 2000 Spectrophotometer (Thermo Scientific, Wilmington, DE, USA), and equal amounts of total RNA from the six groups were further pooled for mRNA library generation constructed by Shanghai Majorbio Bio-pharm Technology Co., Ltd. (Shanghai, China).

Illumina TruseqTM RNA Sample Prep kit (Illumina, San Diego, CA, USA) was used to create mRNA library, according to the manufacturer’s instructions. Briefly, 1 μg pooled total RNA enriched by magnetic beads with oligo dT was broken into 300 bp mRNA fragments with fragmentation buffer. Then, these mRNA fragments were reverse transcribed into cDNA using random primers. cDNA was then ligated with 3′ and 5′ adapters and used for PCR amplification. Then, the amplified cDNA was purified on 6% Novex TBE PAGE gels and evaluated by Picogreen assay (Life Technologies, Carlsbad, CA, USA). Additionally, sequencing was performed on an Illumina Novaseq 6000 machine, using proven Illumina next-generation sequencing (NGS) technology. In addition to being a highly sensitive and accurate means of quantifying gene expression, mRNA-Seq could identify both known and novel transcript isoforms and thus provide transcript abundance for more accurate, comprehensive analysis [82].

### 4.4. Principal Component Analysis

To test whether the mRNA-Seq data were robust, we conducted principal component analysis (PCA) on the outcome of mRNA-Seq, using SIMCA v14.0 (MKS Data Analytics Solutions, Umea, Sweden) as described previously [83]. PCA is mathematically defined as an orthogonal linear transformation that transforms the multivariable data to a new coordinate system. In the new coordinate system, the largest variance by the projection of the data is presented in the first coordinate, called the first principal component (PC1). The second largest variance is subsequently projected onto the second coordinate. This reduces the dimensionality of the data while retaining most of the variation in the dataset. Hence, samples can be plotted to visually assess similarities and differences between samples and to determine whether or not the samples can be grouped. In this study, the differences between the three repeated experiments for ASMCs cultured in either high-stretch or static conditions were used as input variables for the PCA. The data were mean-centered and auto-scaled to unit variance, and 7-fold cross-validations were used to determine the number of significant PCA components.

### 4.5. Differential Expression Analysis of mRNAs in Cultured ASMCs

Signaling molecules in ASMCs may change in either expression, activation or distribution in response to high stretch. However, in a whole genome-wide analysis, the change in gene expression is the easiest to detect. Therefore, we proposed to identify the differentially expressed genes from RNA-Seq data of ASMCs cultured between high-stretch and static conditions as a key to understanding high-stretch-induced signaling response in ASMCs. With the increasing popularity of mRNA-Seq technology, many methods have been developed for analysis of differential gene expression from these data. Although there is no consensus about which is the most appropriate among all the available methods, we used DESeq2 in this study considering its high specificity and true positive rate [77,78].

More specifically, the differential expression of mRNAs in ASMCs cultured in either static or high-stretch conditions was analyzed with the RNA-Seq data on the online platform of Majorbio Cloud Platform (www.majorbio.com, access on 3 September 2022), which is available via customer account [81,84]. The mRNA abundance was quantified by normalizing raw read counts based on transcripts per million (TPM) method. Since many genes in low abundance might not play essential roles in physiological processes even if they could respond to stretch with high fold change, in this study, we filtered out the mRNAs with TPM-standardized counts < 100 to focus on the mRNAs in high abundance. The differential expression of mRNAs in ASMCs cultured in high-stretch condition versus static condition was obtained by analyzing the expression matrix in the R environment using DESeq2 software package. The *p* value was calculated based on a negative binomial distribution model and adjusted by the Benjamini–Hochberg method. A change of 2-fold was used as the cut-off value in order to identify differentially expressed mRNAs (DE-mRNAs) (*p* < 0.05, count ≥ 100) in ASMCs cultured in high-stretch condition as compared to those in static condition [85].

### 4.6. Bioinformatics Analysis

Once the DE-mRNAs were identified, the stretch-induced enrichment in gene functions and signaling pathways including biological processes (BP), molecular functions (MF) as well as cellular components (CC) associated with these DE-mRNAs were further evaluated with Kyoto Encyclopedia of Genes and Genomes (KEGG) pathway analysis and Gene Ontology (GO) analysis, performed by the Database for Annotation, Visualization and Integrated Discovery database (DAVID 6.8, https://david.ncifcrf.gov/, free online access for any user, access on 14 September 2022), respectively, and false discovery rate (FDR) < 0.05 was considered significant [86,87].

DAVID, as well as a number of other tools such as GoMiner, GOstat, Onto-express, GoToolBox, FatiGO, GFINDer, GOBar and GSEA, address various aspects of the challenge of functional enrichment analysis and annotation from large gene lists. Although each tool has its distinct features, they all adopt a common strategy to systematically map interesting genes to the associated biological annotations (e.g., gene ontology terms) and then statistically highlight the most enriched biological annotation. As compared with other tools, DAVID has some unique features to significantly expand its knowledge base and enhance its discovery capabilities, such as an integrated biological knowledgebase, advanced modular enrichment algorithms, and powerful exploratory ability in an integrated data-mining environment, including small molecule–gene interaction from PubChem, drug–gene interaction from DrugBank, tissue expression information from human protein map, disease information from DisGeNET, and pathways from WikiPathways and PathBank [88,89].

### 4.7. Protein–Protein Interaction Analysis and Hub Gene Identification

We then further analyzed the relationships of protein–protein interaction (PPI) between the coding proteins of DE-mRNAs by using the Search Tool for the Retrieval of Interacting Genes/Proteins (STRING) (https://string-db.org/, free online access for any user, access on 20 September 2022), which is a biological database designed to construct a PPI network of genes based on the known and predicted PPIs that are obtained by various methods (experiments, databases, co-expression, neighborhood, gene fusion, and co-occurrence) [90]. The PPI network of DE-mRNAs in this study was constructed with a confidence score ≥ 0.90. A visual PPI network of the DE-mRNAs was constructed using Cytoscape 3.6.1 software.

The hub genes were screened according to interaction degrees ≥ 5 [91]. Subsequently, enrichment analysis of the hub genes including GO and KEGG was performed by DAVID.

### 4.8. Quantitative PCR Analysis of mRNA Expression

ASMCs cultured under the high-stretch or static conditions with or without pretreatment of TUDCA (ER stress inhibitor) were evaluated for mRNA expression levels of genes associated with ER stress and inflammation, which could verify whether ER stress mediated the effects of high stretch on ASMCs via ER stress signaling and airway inflammation [50]. The mRNA expression level was measured by quantitative PCR (qPCR) with the associated primers purchased from General Biosystems (Anhui, China), as shown in Table S5 [92,93,94,95,96,97,98,99,100,101,102,103,104]. These primer sequences used in this study are all obtained from the literature and then checked by NCBI Primer-BLAST (https://www.ncbi.nlm.nih.gov/tools/primer-blast/, freely available for any user, accessed on 5 October 2022). Briefly, total RNA was purified using TRI Reagent RNA Isolation Reagent (#T9424, Sigma-Aldrich, St. Louis, MO, USA), and the extracted RNA was quantified using Nanodrop 2000 Spectrophotometer (Thermo Scientific, Willmington, DE, USA). For mRNA quantification, 500 ng total RNA was used to generate 1st strand cDNA using the Revert Aid First Strand cDNA Synthesis Kit (#K1622, Thermo Scientific). Quantitative real-time PCR (qPCR) was performed with PowerUp SYBR Green Master Mix (#A25742, Applied Biosystems, Foster City, CA, USA) using the StepOne real-time PCR system (Applied Biosystems) at 50 °C for 2 min, 95 °C for 2 min, followed by 40 cycles of 95 °C for 15 s, 55 °C for 15 s and 72 °C for 60 s. The reaction system (10 µL) contained 1 µL of cDNA in triplicates according to the manufacturer’s instructions. Calibration and normalization were performed using the 2^− ∆∆CT^ method, where ∆CT = CT (target gene) − CT (reference gene) and ∆∆CT = ∆CT (experiment groups) − ∆CT (control groups). Fold changes in mRNA expression of different genes were calculated as the ratio of experiment groups to the control groups from the resulting 2^−∆∆CT^ values from three independent experiments.

### 4.9. Statistical Analysis

Statistical analysis was performed by using GraphPad Prism 8.0 (Graph Pad Software, San Diego, CA, USA). Data were reported as means ± S.E.M, and group size (n) represents the number of experiments. Comparisons of means between 2 groups were performed by unpaired Student’s test. Comparisons of means among three or more groups were performed by one-way analysis of variance (ANOVA) followed by a post hoc test (Tukey’s HSD method) or two-way ANOVA followed by the Tukey’s HSD method. The significance of the mean comparisons is represented by asterisks (* *p* <0.05; ** *p* <0.01).

## 5. Conclusions

Overall, we demonstrated from a perspective of whole genome-wide mRNA expression that high stretch activated ER stress and related inflammation signaling in cultured ASMCs, and selective inhibition of ER stress reduced or even completely abolished these responses. These results collectively suggest that ER stress may play essential roles in mediating cellular responses to high stretch and ultimately contribute to high-stretch-induced lung injury. Therefore, it may be a novel strategy to target ER stress and associated biomarkers and factors for timely diagnosis and therapeutic intervention to improve outcomes in treating high-stretch-related diseases such as VILI.

## Figures and Tables

**Table 1:** Top sixteen highest degree nodes.

Node	HSPA5	HSP90B1	CANX	PDIA6	CALR	PPIB	XBP1	P4HB	PDIA3	PDIA4	ATF6	MANF	SEC61A1	RPN2	EIF2AK3	ATF4
Degree	12	12	11	9	9	8	8	7	7	6	6	6	6	6	5	5
Expression change	up	up	up	up	up	up	up	up	up	up	up	up	up	up	up	up

## Data Availability

All the data generated or analyzed during this study are included in this published article.

## Supplementary Materials

The following supporting information can be downloaded at: https://www.mdpi.com/article/10.3390/ijms24043811/s1.
