# Supplementary material for: Mechanical Ventilation-Related High Stretch Mainly Induces Endoplasmic Reticulum Stress and Thus Mediates Inflammation Response in Cultured Human Primary Airway Smooth Muscle Cells
Source: Int J Mol Sci. 2023 Feb 14;24(4):3811. doi: 10.3390/ijms24043811 (PMC9958795; doi:10.3390/ijms24043811)
Supplement: Supplementary file 1 [file ijms-24-03811-s001.zip › ijms-2086465-supplementary-Table S1.pdf]

Table S1: Differently expressed mRNAs (DE-mRNA) with count ≥ 100 ranked with Log_2FC

No.	Name	Gene description	Log_2FC (stretch/static)	Regulate
1	GDF15	growth differentiation factor 15	3.91	up
2	ATF3	activating transcription factor 3	3.59	up
3	ERN1	endoplasmic reticulum to nucleus signaling 1	3.30	up
4	PDIA4	protein disulfide isomerase family A member 4	2.97	up
5	HSPA5	heat shock protein family A (Hsp70) member 5	2.91	up
6	MANF	mesencephalic astrocyte derived neurotrophic factor	2.85	up
7	HSP90B1	heat shock protein 90 beta family member 1	2.35	up
8	PDIA6	protein disulfide isomerase family A member 6	2.13	up
9	CANX	calnexin	1.97	up
10	PDIA3	protein disulfide isomerase family A member 3	1.94	up
11	SQSTM1	sequestosome 1	1.71	up
12	AKR1B1	aldo-keto reductase family 1 member B	1.71	up
13	CALR	calreticulin	1.64	up
14	CTSD	cathepsin D	1.58	up
15	HM13	histocompatibility minor 13	1.56	up
16	EIF2AK3	eukaryotic translation initiation factor 2 alpha kinase 3	1.51	up
17	ATP6V0 B	ATPase H+ transporting V0 subunit b	1.38	up
18	RPN1	ribophorin I	1.31	up
19	NQO1	NAD(P)H quinone dehydrogenase 1	1.26	up

20	FKBP2	FKBP prolyl isomerase 2	1.24	up
21	GYPC	glycophorin C (Gerbich blood group)	1.18	up
22	MT2A	metallothionein 2A	1.15	up
23	OS9	OS9 endoplasmic reticulum lectin	1.13	up
24	RPN2	ribophorin II	1.10	up
25	TMED2	transmembrane p24 trafficking protein 2	1.08	up
26	ATF6	activating transcription factor 6	1.08	up
27	LAMP1	lysosomal associated membrane protein 1	1.07	up
28	PSAP	prosaposin	1.05	up
29	P4HB	prolyl 4-hydroxylase subunit beta	1.04	up
30	SEC61A1	SEC61 translocon subunit alpha 1	1.01	up
31	PPIB	peptidylprolyl isomerase B	1.01	up
32	DDOST	dolichyl-diphosphooligosaccharide--protein glycosyltransferase non-catalytic subunit	1.01	up
33	XBP1	X-box binding protein 1	1.00	up
34	ATF4	activating transcription factor 4	1.00	up
35	PFDN5	prefoldin subunit 5	-1.00	down
36	ATP5MF	ATP synthase membrane subunit f	-1.00	down
37	ILK	integrin linked kinase	-1.00	down
38	NME1	NME/NM23 nucleoside diphosphate kinase 1	-1.01	down
39	SRI	sorcin	-1.01	down
40	MXRA8	matrix remodeling associated 8	-1.02	down
41	ATP5IF1	ATP synthase inhibitory factor subunit 1	-1.02	down
42	MZT2A	mitotic spindle organizing protein 2A	-1.03	down
43	CFL1	cofilin 1	-1.04	down
44	UQCRQ	ubiquinol-cytochrome c reductase complex III	-1.04	down

		subunit VII		
45	GSTP1	glutathione S-transferase pi 1	-1.04	down
46	HNRNP A1	heterogeneous nuclear ribonucleoprotein A1	-1.05	down
47	ATP5F1C	ATP synthase F1 subunit gamma	-1.05	down
48	PHB2	prohibitin 2	-1.05	down
49	COL1A2	collagen type I alpha 2 chain	-1.06	down
50	LSM7	LSM7 homolog, U6 small nuclear RNA and mRNA degradation associated	-1.06	down
51	TMSB10	thymosin beta 10	-1.06	down
52	HMGB1	high mobility group box 1	-1.06	down
53	TAGLN2	transgelin 2	-1.06	down
54	ELOB	elongin B	-1.07	down
55	CALM2	calmodulin 2	-1.08	down
56	IFI27L2	interferon alpha inducible protein 27 like 2	-1.08	down
57	PHPT1	phosphohistidine phosphatase 1	-1.09	down
58	ARF5	ADP ribosylation factor 5	-1.09	down
59	FLNA	filamin A	-1.09	down
60	RARRES 2	retinoic acid receptor responder 2	-1.10	down
61	S100A13	S100 calcium binding protein A13	-1.12	down
62	NDUFS5	NADH:ubiquinone oxidoreductase subunit S5	-1.12	down
63	ROMO1	reactive oxygen species modulator 1	-1.12	down
64	SEPTIN1 1	septin 11	-1.14	down
65	TPM4	tropomyosin 4	-1.16	down

66	ATP5MC3	ATP synthase membrane subunit c locus 3	-1.18	down
67	S100A10	S100 calcium binding protein A10	-1.19	down
68	COTL1	coactosin like F-actin binding protein 1	-1.20	down
69	LDHA	lactate dehydrogenase A	-1.21	down
70	LMNA	lamin A/C	-1.22	down
71	SUB1	SUB1 regulator of transcription	-1.21	down
72	CCDC85B	coiled-coil domain containing 85B	-1.22	down
73	CALM3	calmodulin 3	-1.23	down
74	TUBB	tubulin beta class I	-1.26	down
75	LTBP4	latent transforming growth factor beta binding protein 4	-1.32	down
76	TPI1	triosephosphate isomerase 1	-1.34	down
77	CSRP1	cysteine and glycine rich protein 1	-1.34	down
78	IGFBP4	insulin like growth factor binding protein 4	-1.35	down
79	HSPB1	heat shock protein family B (small) member 1	-1.35	down
80	RTL8C	retrotransposon Gag like 8C	-1.37	down
81	VAMP5	vesicle associated membrane protein 5	-1.38	down
82	TUBB6	tubulin beta 6 class V	-1.39	down
83	CYTOR	cytoskeleton regulator RNA	-1.40	down
84	PPDPF	pancreatic progenitor cell differentiation and proliferation factor	-1.42	down
85	ANXA2	annexin A2	-1.50	down
86	TMEM256	transmembrane protein 256	-1.52	down

87	NME4	NME/NM23 nucleoside diphosphate kinase 4	-1.52	down
88	CAVIN1	caveolae associated protein 1	-1.54	down
89	ACTA2	actin alpha 2, smooth muscle	-1.54	down
90	LGALS1	galectin 1	-1.55	down
91	IMPDH2	inosine monophosphate dehydrogenase 2	-1.56	down
92	PDLIM2	PDZ and LIM domain 2	-1.59	down
93	S100A4	S100 calcium binding protein A4	-1.60	down
94	SPARC	secreted protein acidic and cysteine rich	-1.63	down
95	MDK	midkine	-1.67	down
96	CNN2	calponin 2	-1.71	down
97	FSTL1	follistatin like 1	-1.74	down
98	TUBA1C	tubulin alpha 1c	-1.75	down
99	GAS6	growth arrest specific 6	-1.88	down
100	IFITM2	interferon induced transmembrane protein 2	-1.90	down
101	TPM2	tropomyosin 2	-2.02	down
102	RNA5S13	RNA, 5S ribosomal 13	-2.02	down
103	ACTG1	actin gamma 1	-2.13	down
104	MYL6	myosin light chain 6	-2.21	down
105	COL1A1	collagen type I alpha 1 chain	-2.32	down
106	DCN	decorin	-2.33	down
107	MYL9	myosin light chain 9	-2.41	down
108	TUBA1B	tubulin alpha 1b	-2.76	down
109	RNY1	RNA, Ro60-associated Y1	-2.94	down
110	TAGLN	transgelin	-3.18	down
111	TUBA1A	tubulin alpha 1a	-3.39	down
