# Supplementary material for: Mechanical Ventilation-Related High Stretch Mainly Induces Endoplasmic Reticulum Stress and Thus Mediates Inflammation Response in Cultured Human Primary Airway Smooth Muscle Cells
Source: Int J Mol Sci. 2023 Feb 14;24(4):3811. doi: 10.3390/ijms24043811 (PMC9958795; doi:10.3390/ijms24043811)
Supplement: Supplementary file 1 [file ijms-24-03811-s001.zip › ijms-2086465-supplementary-Table S2.pdf]

Table S2: Enriched KEGG pathway of target DE-mRNAs

ID	Term	Count	FDR	Genes
hsa04141	Protein processing in endoplasmic reticulum	16	9.23E-10	PDIA3, XBP1, RPN2, HSPA5, RPN1, PDIA6, DDOST, PDIA4, HSP90B1, SEC61A1, OS9, CANX, P4HB, CALR, ATF6, ATF4
hsa05014	Amyotrophic lateral sclerosis	16	1.08E-05	XBP1, HSPA5, TUBB, ATP5MC3, ATP5F1C, ACTG1, TUBA1C, TUBA1B, TUBB6, TUBA1A, UQCRQ, NDUFS5, HNRNPA1, ATF6, SQSTM1, ATF4
hsa05022	Pathways of neurodegeneration - multiple diseases	16	0.00019	XBP1, HSPA5, TUBB, ATP5MC3, ATP5F1C, TUBA1C, TUBA1B, TUBB6, TUBA1A, UQCRQ, NDUFS5, CALM3, CALM2, ATF6, SQSTM1, ATF4
hsa05012	Parkinson disease	15	2.12E-06	XBP1, HSPA5, TUBB, ATP5MC3, ATP5F1C, TUBA1C, TUBA1B, TUBB6, TUBA1A, UQCRQ, NDUFS5, CALM3, CALM2, ATF6, ATF4
hsa05010	Alzheimer disease	14	0.000342	XBP1, TUBB, ATP5MC3, ATP5F1C, TUBA1C, TUBA1B, TUBB6, TUBA1A, UQCRQ, NDUFS5, CALM3, CALM2, ATF6, ATF4
hsa04145	Phagosome	11	1.64E-05	TUBA1C, SEC61A1, ATP6V0B, TUBA1B, TUBB6, TUBA1A, LAMP1, TUBB, CANX, CALR, ACTG1
hsa05132	Salmonella infection	11	0.00078	TUBA1C, TUBA1B, TUBB6, TUBA1A, ANXA2, TUBB, FLNA, MYL9, HSP90B1, ACTG1, S100A10
hsa05020	Prion disease	11	0.001487	TUBA1C, TUBA1B, TUBB6, TUBA1A, HSPA5, UQCRQ, NDUFS5, TUBB, ATP5MC3, ATP5F1C, ATF4
hsa05016	Huntington disease	9	0.04731	TUBA1C, TUBA1B, TUBB6, TUBA1A, UQCRQ, NDUFS5, TUBB, ATP5MC3, ATP5F1C

hsa04210	Apoptosis	7	0.015242	TUBA1C, TUBA1B, TUBA1A, LMNA, CTSD, ATF4, ACTG1
hsa05418	Fluid shear stress and atherosclerosis	7	0.015372	NQO1, GSTP1, CALM3, CALM2, SQSTM1, HSP90B1, ACTG1
hsa05415	Diabetic cardiomyopathy	7	0.067966	COL1A1, COL1A2, UQCRQ, NDUFS5, ATP5MC3, ATP5F1C, CTSD
hsa05417	Lipid and atherosclerosis	7	0.07865	XBP1, HSPA5, CALM3, CALM2, ATF6, HSP90B1, ATF4
hsa00190	Oxidative phosphorylation	6	0.059158	ATP6V0B, UQCRQ, NDUFS5, ATP5MC3, ATP5F1C, ATP5MF
hsa04530	Tight junction	6	0.110851	TUBA1C, TUBA1B, MYL6, TUBA1A, MYL9, ACTG1
hsa05130	Pathogenic Escherichia coli infection	6	0.18283	TUBA1C, TUBA1B, TUBB6, TUBA1A, TUBB, ACTG1
hsa04510	Focal adhesion	6	0.18283	COL1A1, COL1A2, ILK, FLNA, MYL9, ACTG1
hsa05170	Human immunodeficiency virus 1 infection	6	0.191193	PDIA3, CFL1, CALM3, ELOB, CALR, CALM2
hsa04714	Thermogenesis	6	0.221394	UQCRQ, NDUFS5, ATP5MC3, ATP5F1C, ATP5MF, ACTG1
