# Supplementary material for: Mechanical Ventilation-Related High Stretch Mainly Induces Endoplasmic Reticulum Stress and Thus Mediates Inflammation Response in Cultured Human Primary Airway Smooth Muscle Cells
Source: Int J Mol Sci. 2023 Feb 14;24(4):3811. doi: 10.3390/ijms24043811 (PMC9958795; doi:10.3390/ijms24043811)
Supplement: Supplementary file 1 [file ijms-24-03811-s001.zip › ijms-2086465-supplementary-Table S3.pdf]

Table S3: GO enrichment terms of target DE-mRNAs in ASMCs

ID	Term	Category	Count	PValue	FDR
GO:0043066	Negative regulation of apoptotic process	BP	12	9.84E-05	0.011187
GO:0006457	Protein folding	BP	11	1.69E-08	5.76E-06
GO:0010628	Positive regulation of gene expression	BP	9	0.00558	0.184145
GO:0030154	Cell differentiation	BP	9	0.026541	0.493668
GO:0030968	Endoplasmic reticulum unfolded protein response	BP	8	9.75E-09	4.99E-06
GO:0034976	Response to endoplasmic reticulum stress	BP	8	2.88E-07	6.99E-05
GO:0042493	Response to drug	BP	8	0.000893	0.057119
GO:0043065	Positive regulation of apoptotic process	BP	7	0.007645	0.217243
GO:0034975	Protein folding in endoplasmic reticulum	BP	6	1.62E-09	1.66E-06
GO:1900026	Positive regulation of substrate adhesion-dependent cell spreading	BP	6	3.32E-06	0.000543
GO:0070527	Platelet aggregation	BP	6	3.72E-06	0.000543
GO:0001934	Positive regulation of protein phosphorylation	BP	6	0.0049	0.17901
GO:0050821	Protein stabilization	BP	6	0.006068	0.188119
GO:0030335	Positive regulation of cell migration	BP	6	0.011252	0.28075
GO:0015031	Protein transport	BP	6	0.081231	0.81271
GO:1990440	Positive regulation of transcription from RNA polymerase II promoter in response to endoplasmic reticulum stress	BP	5	3.42E-07	6.99E-05
GO:0007017	Microtubule-based process	BP	5	5.64E-05	0.007207
GO:0030433	Ubiquitin-dependent ERAD pathway	BP	5	0.00087	0.057119
GO:0007517	Muscle organ development	BP	5	0.001902	0.087005
GO:0000226	Microtubule cytoskeleton organization	BP	5	0.00544	0.184145
GO:0000278	Mitotic cell cycle	BP	5	0.008819	0.23741
GO:0070062	Extracellular exosome	CC	47	3.05E-18	7.65E-16
GO:0005737	Cytoplasm	CC	45	0.00062	0.007069
GO:0005829	Cytosol	CC	44	0.000861	0.009002
GO:0005634	Nucleus	CC	44	0.004547	0.034586
GO:0016020	Membrane	CC	32	2.05E-06	5.14E-05

GO:0005615	Extracellular space	CC	30	6.56E-08	3.29E-06
GO:0005576	Extracellular region	CC	30	4.88E-07	1.53E-05
GO:0005783	Endoplasmic reticulum	CC	24	1.06E-08	6.65E-07
GO:0005788	Endoplasmic reticulum lumen	CC	18	2.54E-13	3.18E-11
GO:0032991	Macromolecular complex	CC	16	2.63E-06	5.99E-05
GO:0005789	Endoplasmic reticulum membrane	CC	16	0.000272	0.004128
GO:0005925	Focal adhesion	CC	14	2.38E-07	8.54E-06
GO:0048471	Perinuclear region of cytoplasm	CC	14	0.00013	0.002174
GO:0005739	Mitochondrion	CC	14	0.030075	0.167751
GO:0009986	Cell surface	CC	13	9.57E-05	0.001716
GO:0042470	Melanosome	CC	12	4.58E-12	3.83E-10
GO:0005856	Cytoskeleton	CC	11	0.000432	0.005705
GO:0031982	Vesicle	CC	8	3.02E-05	0.000582
GO:0005743	Mitochondrial inner membrane	CC	8	0.009479	0.064379
GO:0001725	Stress fiber	CC	7	1.92E-06	5.14E-05
GO:0045121	Membrane raft	CC	7	0.001784	0.015993
GO:0005515	Protein binding	MF	95	8.28E-12	2.03E-09
GO:0042802	Identical protein binding	MF	28	1.38E-07	1.69E-05
GO:0003723	RNA binding	MF	25	4.43E-07	2.71E-05
GO:0005509	Calcium ion binding	MF	18	3.64E-07	2.71E-05
GO:0042803	Protein homodimerization activity	MF	14	9.18E-05	0.00225
GO:0031625	Ubiquitin protein ligase binding	MF	12	6.04E-07	2.96E-05
GO:0046982	Protein heterodimerization activity	MF	9	0.000763	0.013358
GO:0019901	Protein kinase binding	MF	9	0.005484	0.06718
GO:0003779	Actin binding	MF	8	0.002094	0.030185
GO:0005525	GTP binding	MF	8	0.005131	0.066158
GO:0005200	Structural constituent of cytoskeleton	MF	7	2.15E-05	0.000835
GO:0002020	Protease binding	MF	7	2.38E-05	0.000835
GO:0051082	Unfolded protein binding	MF	7	6.71E-05	0.002054
GO:0019904	Protein domain specific binding	MF	7	0.002673	0.036383
GO:0019899	Enzyme binding	MF	7	0.017056	0.167854

CC: cellular component; BP: biological process; MF: molecular function.
