# Supplementary material for: Mechanical Ventilation-Related High Stretch Mainly Induces Endoplasmic Reticulum Stress and Thus Mediates Inflammation Response in Cultured Human Primary Airway Smooth Muscle Cells
Source: Int J Mol Sci. 2023 Feb 14;24(4):3811. doi: 10.3390/ijms24043811 (PMC9958795; doi:10.3390/ijms24043811)
Supplement: Supplementary file 1 [file ijms-24-03811-s001.zip › ijms-2086465-supplementary-Table S4.pdf]

Table S4: PPI network of target DE-mRNAs

#node1	node2	node1_string_id	node2_string_id	homology	coexpression	experimentally_determined_interaction	database_annotated	automated_textmining	combined_score
ACTG1	CFL1	9606.ENSP00000458162	9606.ENSP00000432660	0	0.279	0.798	0.8	0.752	0.991
ANXA2	S100A4	9606.ENSP00000346032	9606.ENSP00000357705	0	0.199	0.835	0	0.995	0.999
ANXA2	S100A10	9606.ENSP00000346032	9606.ENSP00000357801	0	0.714	0.901	0.9	0.989	0.999
ATF3	ATF4	9606.ENSP00000344352	9606.ENSP00000336790	0	0.063	0.704	0.8	0.712	0.981
ATF4	XBP1	9606.ENSP00000336790	9606.ENSP00000216037	0	0.073	0.182	0	0.887	0.907
ATF4	EIF2AK3	9606.ENSP00000336790	9606.ENSP00000307235	0	0	0	0.8	0.793	0.957
ATF4	HSPA5	9606.ENSP00000336790	9606.ENSP00000324173	0	0.065	0	0.9	0.881	0.987
ATF4	ATF6	9606.ENSP00000336790	9606.ENSP00000356919	0	0	0	0	0.904	0.904
ATF6	XBP1	9606.ENSP00000356919	9606.ENSP00000216037	0	0	0.458	0.9	0.973	0.998
ATF6	HSP90B1	9606.ENSP00000356919	9606.ENSP00000299767	0	0.063	0	0.9	0.859	0.985
ATF6	EIF2AK3	9606.ENSP00000356919	9606.ENSP00000307235	0	0.056	0	0	0.922	0.924
ATF6	CALR	9606.ENSP00000	9606.ENSP00000	0	0.062	0	0.9	0.903	0.99

		356919	320866						
ATF6	HSPA5	9606.ENSP00000356919	9606.ENSP00000324173	0	0.062	0.345	0.8	0.995	0.999
ATF6	ERN1	9606.ENSP00000356919	9606.ENSP00000401445	0	0.062	0	0	0.933	0.934
ATP5C1	ATP5G3	9606.ENSP00000349142	9606.ENSP00000284727	0	0.866	0.742	0.6	0.724	0.996
ATP5C1	ATP5J2	9606.ENSP00000349142	9606.ENSP00000292475	0	0.881	0.775	0.9	0.566	0.998
ATP5C1	UQCRQ	9606.ENSP00000349142	9606.ENSP00000367939	0	0.861	0.372	0	0.348	0.938
ATP5G3	UQCRQ	9606.ENSP00000284727	9606.ENSP00000367939	0	0.856	0	0	0.524	0.928
ATP5G3	ATP5J2	9606.ENSP00000284727	9606.ENSP00000292475	0	0.772	0.787	0.8	0.551	0.995
ATP5J2	UQCRQ	9606.ENSP00000292475	9606.ENSP00000367939	0	0.962	0.271	0	0.446	0.983
CALR	CANX	9606.ENSP00000320866	9606.ENSP00000247461	0.852	0.325	0.386	0.8	0.994	0.923
CALR	PDIA4	9606.ENSP00000320866	9606.ENSP00000286091	0	0.881	0.358	0	0.786	0.982
CALR	HSP90B1	9606.ENSP00000320866	9606.ENSP00000299767	0	0.954	0.786	0.3	0.919	0.999
CALR	PPIB	9606.ENSP00000320866	9606.ENSP00000300026	0	0.643	0.143	0	0.851	0.95
CALR	PDIA	9606.ENSP00000	9606.ENSP00000	0	0.928	0.962	0.9	0.995	0.999

	3	320866	300289						
CALR	PDIA 6	9606.ENSP00000 320866	9606.ENSP00000 385385	0	0.905	0.394	0	0.656	0.978
CALR	HSPA 5	9606.ENSP00000 320866	9606.ENSP00000 324173	0	0.88	0.789	0.65	0.894	0.998
CALR	P4HB	9606.ENSP00000 320866	9606.ENSP00000 327801	0	0.854	0.891	0	0.992	0.999
CAN X	RPN2	9606.ENSP00000 247461	9606.ENSP00000 237530	0	0.516	0.819	0	0.429	0.945
CAN X	SEC61 A1	9606.ENSP00000 247461	9606.ENSP00000 243253	0	0.509	0.413	0	0.851	0.953
CAN X	OS9	9606.ENSP00000 247461	9606.ENSP00000 318165	0	0.299	0	0	0.878	0.911
CAN X	RPN1	9606.ENSP00000 247461	9606.ENSP00000 296255	0	0.342	0.802	0	0.692	0.956
CAN X	PDIA 4	9606.ENSP00000 247461	9606.ENSP00000 286091	0	0.51	0.078	0	0.918	0.96
CAN X	DDOS T	9606.ENSP00000 247461	9606.ENSP00000 399457	0	0.766	0.829	0	0.392	0.973
CAN X	P4HB	9606.ENSP00000 247461	9606.ENSP00000 327801	0	0.532	0.078	0	0.954	0.978
CAN X	HSP9 0B1	9606.ENSP00000 247461	9606.ENSP00000 299767	0	0.843	0.425	0	0.9	0.99
CAN X	HSPA 5	9606.ENSP00000 247461	9606.ENSP00000 324173	0	0.7	0.69	0.65	0.963	0.998
CAN	PDIA	9606.ENSP00000	9606.ENSP00000	0	0.57	0.689	0.6	0.993	0.999

X	3	247461	300289						
CFL1	TPI1	9606.ENSP00000432660	9606.ENSP00000229270	0	0.169	0.27	0	0.89	0.927
COL1A1	DCN	9606.ENSP00000225964	9606.ENSP00000052754	0	0.864	0.241	0	0.636	0.959
COL1A1	SPARC	9606.ENSP00000225964	9606.ENSP00000231061	0	0.94	0.515	0	0.669	0.989
COL1A1	COL1A2	9606.ENSP00000225964	9606.ENSP00000297268	0.958	0.99	0.559	0.9	0.954	0.999
COL1A2	DCN	9606.ENSP00000297268	9606.ENSP00000052754	0	0.927	0.241	0	0.567	0.974
COL1A2	SPARC	9606.ENSP00000297268	9606.ENSP00000231061	0	0.973	0.239	0	0.563	0.99
CTSD	PSAP	9606.ENSP00000236671	9606.ENSP00000378394	0	0.542	0.497	0	0.711	0.928
DDOST	RPN2	9606.ENSP00000399457	9606.ENSP00000237530	0	0.991	0.995	0.9	0.992	0.999
DDOST	SEC61A1	9606.ENSP00000399457	9606.ENSP00000243253	0	0.806	0.38	0.6	0.722	0.984
DDOST	RPN1	9606.ENSP00000399457	9606.ENSP00000296255	0	0.991	0.996	0.9	0.982	0.999
EIF2AK3	XBP1	9606.ENSP00000307235	9606.ENSP00000216037	0	0.085	0	0	0.934	0.937
EIF2AK3	FLNA	9606.ENSP00000307235	9606.ENSP00000358866	0	0.062	0	0	0.909	0.911
EIF2A	HSPA	9606.ENSP00000	9606.ENSP00000	0	0.083	0.877	0.8	0.994	0.999

K3	5	307235	324173						
ERN1	XBP1	9606.ENSP00000401445	9606.ENSP00000216037	0	0	0.14	0.8	0.986	0.997
ERN1	HSPA5	9606.ENSP00000401445	9606.ENSP00000324173	0	0	0.952	0.8	0.994	0.999
FKBP2	PPIB	9606.ENSP00000378046	9606.ENSP00000300026	0	0.138	0.739	0	0.668	0.918
FLNA	TAGLN2	9606.ENSP00000358866	9606.ENSP00000357076	0	0.208	0.167	0.9	0.307	0.948
FLNA	MANF	9606.ENSP00000358866	9606.ENSP00000432799	0	0	0	0.9	0.069	0.903
HSP90B1	RPN2	9606.ENSP00000299767	9606.ENSP00000237530	0	0.849	0.621	0	0.308	0.957
HSP90B1	PDIA4	9606.ENSP00000299767	9606.ENSP00000286091	0	0.937	0.603	0	0.948	0.998
HSP90B1	MANF	9606.ENSP00000299767	9606.ENSP00000432799	0	0.926	0	0	0.397	0.953
HSP90B1	PPIB	9606.ENSP00000299767	9606.ENSP00000300026	0	0.394	0.55	0.36	0.804	0.961
HSP90B1	PDIA3	9606.ENSP00000299767	9606.ENSP00000300289	0	0.904	0.349	0	0.785	0.985
HSP90B1	OS9	9606.ENSP00000299767	9606.ENSP00000318165	0	0.112	0.883	0	0.944	0.993
HSP90B1	P4HB	9606.ENSP00000299767	9606.ENSP00000327801	0	0.632	0.775	0.36	0.927	0.995
HSP9	PDIA	9606.ENSP00000	9606.ENSP00000	0	0.94	0.727	0.36	0.697	0.996

0B1	6	299767	385385						
HSP90B1	HSPA5	9606.ENSP00000299767	9606.ENSP00000324173	0	0.974	0.753	0.36	0.98	0.999
HSPA5	XBP1	9606.ENSP00000324173	9606.ENSP00000216037	0	0.466	0.282	0	0.937	0.974
HSPA5	SEC61A1	9606.ENSP00000324173	9606.ENSP00000243253	0	0.669	0.644	0	0.887	0.985
HSPA5	PDIA4	9606.ENSP00000324173	9606.ENSP00000286091	0	0.93	0.663	0	0.823	0.995
HSPA5	PPIB	9606.ENSP00000324173	9606.ENSP00000300026	0	0.475	0.734	0.36	0.608	0.96
HSPA5	PDIA3	9606.ENSP00000324173	9606.ENSP00000300289	0	0.72	0.743	0.6	0.858	0.995
HSPA5	OS9	9606.ENSP00000324173	9606.ENSP00000318165	0	0.344	0.733	0	0.868	0.975
HSPA5	SQSTM1	9606.ENSP00000324173	9606.ENSP00000374455	0	0	0.887	0	0.85	0.982
HSPA5	PDIA6	9606.ENSP00000324173	9606.ENSP00000385385	0	0.742	0.759	0.54	0.791	0.993
HSPA5	P4HB	9606.ENSP00000324173	9606.ENSP00000327801	0	0.743	0.744	0.36	0.903	0.995
HSPA5	MANF	9606.ENSP00000324173	9606.ENSP00000432799	0	0.931	0.703	0	0.965	0.999
ILK	MYL9	9606.ENSP00000379975	9606.ENSP00000279022	0	0.129	0.078	0.9	0.396	0.945
LMN	XBP1	9606.ENSP00000	9606.ENSP00000	0	0	0	0.9	0.184	0.914

A		357283	216037						
MANF	PDIA4	9606.ENSP00000432799	9606.ENSP00000286091	0	0.927	0	0	0.434	0.956
MANF	TAGLN2	9606.ENSP00000432799	9606.ENSP00000357076	0	0	0	0.9	0	0.9
MANF	PDIA6	9606.ENSP00000432799	9606.ENSP00000385385	0	0.888	0	0	0.404	0.93
MYL9	TPM2	9606.ENSP00000279022	9606.ENSP00000367542	0	0.532	0.103	0.6	0.602	0.924
NDUFS5	UQCRQ	9606.ENSP00000362060	9606.ENSP00000367939	0	0.929	0.959	0	0.5	0.998
NME1	NME4	9606.ENSP00000337060	9606.ENSP00000219479	0.944	0.076	0.743	0.8	0.757	0.951
OS9	P4HB	9606.ENSP00000318165	9606.ENSP00000327801	0	0.245	0	0.65	0.796	0.941
P4HB	PPIB	9606.ENSP00000327801	9606.ENSP00000300026	0	0.387	0.723	0.9	0.717	0.994
P4HB	PDIA6	9606.ENSP00000327801	9606.ENSP00000385385	0.603	0.609	0.747	0.36	0.813	0.953
PDIA3	RPN2	9606.ENSP00000300289	9606.ENSP00000237530	0	0.85	0.271	0	0.24	0.909
PDIA3	PPIB	9606.ENSP00000300289	9606.ENSP00000300026	0	0.351	0.776	0	0.589	0.935
PDIA3	PDIA6	9606.ENSP00000300289	9606.ENSP00000385385	0.616	0.842	0.368	0	0.814	0.928
PDIA	PDIA	9606.ENSP00000	9606.ENSP00000	0.696	0.876	0.385	0	0.844	0.94

4	6	286091	385385						
PDIA 4	PPIB	9606.ENSP00000 286091	9606.ENSP00000 300026	0	0.322	0.685	0	0.947	0.987
PDIA 6	XBP1	9606.ENSP00000 385385	9606.ENSP00000 216037	0	0.127	0	0.9	0.557	0.957
PDIA 6	PPIB	9606.ENSP00000 385385	9606.ENSP00000 300026	0	0.384	0.74	0.36	0.569	0.95
PTRF	S100A 10	9606.ENSP00000 349541	9606.ENSP00000 357801	0	0.943	0	0	0.079	0.945
RPN1	RPN2	9606.ENSP00000 296255	9606.ENSP00000 237530	0	0.989	0.995	0.9	0.996	0.999
RPN1	SEC61 A1	9606.ENSP00000 296255	9606.ENSP00000 243253	0	0.279	0.107	0.6	0.78	0.936
RPN2	SEC61 A1	9606.ENSP00000 237530	9606.ENSP00000 243253	0	0.525	0.532	0.6	0.728	0.972
SEC61 A1	XBP1	9606.ENSP00000 243253	9606.ENSP00000 216037	0	0.092	0.8	0	0.553	0.911
TPM2	TPM4	9606.ENSP00000 367542	9606.ENSP00000 345230	0.979	0.076	0.627	0.9	0.764	0.963
TUBA 1A	TUBA 1C	9606.ENSP00000 301071	9606.ENSP00000 301072	0.987	0.325	0.129	0.9	0.566	0.936
TUBA 1A	TUBB 6	9606.ENSP00000 301071	9606.ENSP00000 318697	0.919	0.155	0.752	0.8	0.635	0.956
TUBA 1A	TUBA 1B	9606.ENSP00000 301071	9606.ENSP00000 336799	0.987	0.311	0.941	0.3	0.574	0.969
TUBA	TUBB	9606.ENSP00000	9606.ENSP00000	0.92	0.224	0.836	0.8	0.643	0.973

1A		301071	339001						
TUBA	TUBA	9606.ENSP00000	9606.ENSP00000	0.987	0.789	0.877	0.3	0.52	0.98
1B	1C	336799	301072						
TUBA	TUBB	9606.ENSP00000	9606.ENSP00000	0.92	0.123	0.723	0.8	0.634	0.949
1B	6	336799	318697						
TUBA	TUBB	9606.ENSP00000	9606.ENSP00000	0.921	0.96	0.946	0.8	0.569	0.999
1B		336799	339001						
TUBA	TUBB	9606.ENSP00000	9606.ENSP00000	0.921	0.388	0.665	0.8	0.511	0.957
1C		301072	339001						
TUBA	TUBB	9606.ENSP00000	9606.ENSP00000	0.919	0.21	0.746	0.8	0.478	0.958
1C	6	301072	318697						
