# Supplementary material for: Mechanical Ventilation-Related High Stretch Mainly Induces Endoplasmic Reticulum Stress and Thus Mediates Inflammation Response in Cultured Human Primary Airway Smooth Muscle Cells
Source: Int J Mol Sci. 2023 Feb 14;24(4):3811. doi: 10.3390/ijms24043811 (PMC9958795; doi:10.3390/ijms24043811)
Supplement: Supplementary file 1 [file ijms-24-03811-s001.zip › ijms-2086465-supplementary-Table S5.pdf]

Table S5: Gene-specific primers used in this study

Gene name	Primer sequence (5' to 3')	Reference
HSPA5	Forward: GTGGAATGACCCGTCTGTG<br>Reverse: TTGGTTGCTTGGCGTTGG	[92]
EIF2AK3	Forward: AATGCCTGGGACGTGGTGGC<br>Reverse: TGGTGGTGCTTCGAGCCAGG	[93]
ERN1	Forward: TGCTTAAGGACATGGCTACCATCA<br>Reverse: CTGGAACTGCTGGTGCTGGA	[93]
ATF6	Forward: TCCTCGGTCAGTGGACTCTTA<br>Reverse: CTTGGGCTGAATTGAAGGTTTTG	[94]
ATF4	Forward: ATGACCGAAATGAGCTTCCTG<br>Reverse: GCTGGAGAACCCATGAGGT	[95]
DDIT3	Forward: CACCTCCTGGAAATGAAGAGGA<br>Reverse: TGCTTGTGACCTCTGCTGGT	[96]
IL1β	Forward: CTGAGCTCGCCAGTGAAATG<br>Reverse: TGTCCATGGCCACAACAACT	[97]
IL6	Forward: CCAGAGCTGTGCAGATGAGT<br>Reverse: AGTTGTCATGTCCTGCAGCC	[98]
IL8	Forward: GGCAGCCTTCCTGATTTC<br>Reverse: CTCAGCCCTCTTCAAAAACTTC	[98]
IL10	Forward: GACTTTAAGGGTTACCTGGGTTG<br>Reverse: TCACATGCGCCTTGATGTCTG	[100]
GM-CSF	Forward: AATGTTTGACCTCCAGGAGCC<br>Reverse: TCTGGGTTGCACAGGAAGTTT	[101]
TGFβ1	Forward:TGAACCGGCCTTTCCTGCTTCTCATG<br>Reverse: GCGGAAGTCAATGTACAGCTGCCGC	[102]
ACTIN	Forward: GGATGCAGAAGGAGATCACTG<br>Reverse: CGATCCACACGGAGTACTTG	[103]
ADRβ2	Forward: TTCCTCTTTGCATGGAATTTG<br>Reverse: AGAGGAGTGGGGAAGAGTC	[104]

References

92. Liu, Y.P.; Rajamanikham, V.; Baron, M.; Patel, S.; Mathur, S.K.; Schwantes, E.A.; Ober, C.; Jackson, D.J.; Gern, J.E.; Lemanske, R.F., Jr.; et al. Association of ORMDL3 with rhinovirus-induced endoplasmic reticulum stress and type I Interferon responses in human leucocytes. *Clin. Exp. Allergy* **2017**, *47*, 371-382.
93. Garcia-Gonzalez, P.; Fernandez, D.; Gutierrez, D.; Parra-Cordero, M.; Osorio, F. Human cDC1s display constitutive activation of the UPR sensor IRE1. *Eur. J. Immunol.* **2022**, *52*, 1069-1076.
94. Gong, Y.; Li, Q.; Ma, Z.; Jin, T.; Lin, J.; Lv, Q.; Wang, M.; Fu, G.; Xu, S. Downregulation of activating transcription factor 4 attenuates lysophosphatidycholine-induced inflammation via the NF-kappaB pathway. *Eur. J. Pharmacol.* **2021**, *911*, 174457.

95. Chen, H.; Zhang, Y.; Su, H.; Shi, H.; Xiong, Q.; Su, Z. Overexpression of miR-1283 Inhibits Cell Proliferation and Invasion of Glioma Cells by Targeting ATF4. *Oncol. Res.* **2019**, *27*, 325-334.
96. Katharine M, P.G., Xiaoyu An. Peripheral blood monocyte gene expression profile clinically stratifies patients with recent-onset type 1 diabetes. *Diabetes* **2012**, *61*, 1281-1290.
97. Duan, X.J.; Zhang, X.; Li, L.R.; Zhang, J.Y.; Chen, Y.P. MiR-200a and miR-200b restrain inflammation by targeting ORMDL3 to regulate the ERK/MMP-9 pathway in asthma. *Experimental lung research* **2020**, *46*, 321-331.
98. Zhang, Z.; Liang, Z.; Li, H.; Li, C.; Yang, Z.; Li, Y.; She, D.; Cao, L.; Wang, W.; Liu, C.; et al. Perfluorocarbon reduces cell damage from blast injury by inhibiting signal paths of NF-kappaB, MAPK and Bcl-2/Bax signaling pathway in A549 cells. *PLoS One* **2017**, *12*, e0173884.
99. Yanzhang, P. Stretch-induced expression of CYR61 increases the secretion of IL-8 in A549 Cells via the NF-κβ lκβ pathway. *Curr. Med. Sci.* **2018**, *38*, 672-678.
100. Huang, Q.; Hua, H.; Li, W.; Chen, X.; Cheng, L. Simple hypertrophic tonsils have more active innate immune and inflammatory responses than hypertrophic tonsils with recurrent inflammation in children. *J. Otolaryngol. Head Neck Surg.* **2020**, *49*, 35.
101. Hong, Z.; Luo, X.; Cai, C.; Xu, J.; Zhuang, G. Airborne fine particle decreases the cell viability and induces inflammation in human bronchial epithelial cells. *Zhong Nan Da Xue Xue Bao Yi Xue Ban* **2017**, *42*, 1042-1047.
102. Risse, P.A.; Jo, T.; Suarez, F.; Hirota, N.; Tolloczko, B.; Ferraro, P.; Grutter, P.; Martin, J.G. Interleukin-13 inhibits proliferation and enhances contractility of human airway smooth muscle cells without change in contractile phenotype. *Am. J. Physiol. Lung Cell Mol. Physiol.* **2011**, *300*, L958-966.
103. Tassignon, J.; Burny, W.; Dahmani, S.; Zhou, L.; Stordeur, P.; Byl, B.; De Groote, D. Monitoring of cellular responses after vaccination against tetanus toxoid: comparison of the measurement of IFN-gamma production by ELISA, ELISPOT, flow cytometry and real-time PCR. *J. Immunol. Methods.* **2005**, *305*, 188-198.
104. Chen, D.; Xing, W.; Hong, J.; Wang, M.; Huang, Y.; Zhu, C.; Yuan, Y.; Zeng, W. The beta2-adrenergic receptor is a potential prognostic biomarker for human hepatocellular carcinoma after curative resection. *Ann. Surg. Oncol.* **2012**, *19*, 3556-3565.
